# Induced and pre-existing anti-polyethylene glycol antibody in a trial of every 3-week dosing of pegloticase for refractory gout, including in organ transplant recipients

**DOI:** 10.1186/ar4500

**Published:** 2014-03-07

**Authors:** Michael S Hershfield, Nancy J Ganson, Susan J Kelly, Edna L Scarlett, Denise A Jaggers, John S Sundy

**Affiliations:** 1 Duke University Medical Center, Box 3049, Durham, NC 27710, USA; 2 Duke Clinical Research Institute, Durham, NC, USA; 3 Duke-National University of Singapore Graduate Medical School, Singapore, Singapore

## Abstract

**Introduction:**

Pegloticase, a PEGylated recombinant porcine uricase, is approved for treating refractory gout at a dose of 8 mg intravenous (IV) every 2 weeks. However, during phase 1 testing, pharmacokinetics supported less frequent dosing. Also, single doses of pegloticase unexpectedly induced antibodies (Ab) that bound to polyethylene glycol (PEG). We have conducted a phase 2 trial to evaluate every 3-week dosing, and to further define the Ab response to pegloticase. Organ transplant recipients were included, as they are prone to severe gout that is difficult to manage, and because treatment to prevent graft rejection might influence the immune response to pegloticase.

**Methods:**

Plasma uricase activity (pUox), urate concentration (pUA), and clinical response were monitored during up to 5 infusions in 30 patients, including 7 organ transplant recipients. Depending on whether pUA <6 mg/dL was achieved and maintained, patients were classified as non (NR), persistent (PR), or transient (TR) responders. Ab to pegloticase and 10 kDa mPEG were monitored by enzyme linked immunosorbent assay and specificity was further defined.

**Results:**

We observed 17 PR, 12 TR, and 1 NR; 21 patients (16 PR, 5 TR) received all 5 infusions. Over the 15-week trial, pUA in PR averaged 1.0 ± 0.4 mg/dL; T_**½**_ for pUox was approximately 13 days, and area under the curve after dose 5 was approximately 30% higher than after dose 1. PR showed clinical benefit and in some, tophi resolved. In 11 of 12 TR, pUox fell rapidly and hyperuricemia recurred before dose 2. In all TR and NR, loss of response to pegloticase was accompanied by Ab to PEG, which was pre-existing in half of those who had no prior exposure to pegloticase. No PR, and 1 one out of 7 organ transplant recipients, had a sustained Ab response to pegloticase.

**Conclusions:**

Every 3-week dosing is effective and may enhance the utility of pegloticase for treating refractory gout. Ab to PEG, which were pre-existing or induced by treatment, caused rapid loss of efficacy and increased the risk of infusion reactions. Organ transplant recipients can benefit from pegloticase, and may be less prone than non-recipients to developing anti-PEG Ab. Investigation of immunosuppressive strategies to minimize anti-PEG Ab is warranted.

**Trial registration:**

ClincalTrials.gov identifier: NCT00111657

## Introduction

Owing to poorly controlled hyperuricemia, a subset of gout patients reach an advanced stage, typically characterized by debilitating arthropathy and tophi [1-3]. At this late stage, relieving arthritis and eliminating tophi are difficult to achieve, and treatment is often complicated by age- and gout-associated comorbidities, such as hypertension, renal insufficiency, diabetes and vascular disease. In 2010, pegloticase (Krystexxa®), a PEGylated recombinant porcine urate oxidase, was approved as an Orphan Drug specifically for treating refractory gout.

The FDA-approved dose of pegloticase, 8 mg i.v. every 3 weeks, was more effective in maintaining pUA below 6 mg/dL than every four week dosing in phase 3 trials [4,5]. Whether every two week dosing is necessary to meet that target is unclear, as in phase 1 testing, a single infusion of 8 mg maintained pUA at <2 mg/dL for 21 days, although this dose was tested in only four patients [6]. We have conducted the present trial to assess the urate-lowering efficacy of an 8 mg dose of pegloticase infused at three-week intervals. The difference between a two- and a three-week infusion schedule is not trivial, considering that travel to spend most of a day in an infusion clinic is often difficult for patients with advanced gout.

In addition to the optimal infusion schedule, we have investigated two other issues relevant to the utility and safety of pegloticase. One is a hypothetical concern: that pegloticase might exacerbate oxidative stress by depleting uric acid while generating hydrogen peroxide. We have reported separately that oxidative stress status did not worsen in patients during the present trial, as erythrocytes had more than sufficient enzymatic capacity to eliminate the hydrogen peroxide generated from urate oxidation, and to compensate for the loss of urate in maintaining the antioxidant capacity of blood [7].

The second focus of this report is the humoral immune response to pegloticase. During phase 1 testing we found that single doses of pegloticase induced Ab that appeared to recognize PEG [6,8]. This was unexpected, as PEGylation of biologic agents is intended in part to reduce immunogenicity, and PEG itself is not immunogenic [9]. In phase 3 trials, high titer Ab to pegloticase was the principle reason for loss of efficacy, but epitope specificity was not reported [4]. The existence and significance of Ab to PEG in humans is controversial, but of potential importance as PEGylated biologics are used to treat a variety of diseases, including some of interest to rheumatologists [10,11].

The present trial was designed to establish whether anti-PEG Ab is a transient or persistent immune response to pegloticase during repeated dosing over a period sufficient to achieve clinical benefit. We have explored in detail the relationship between Ab evolution and the pharmacokinetics and pharmacodynamics associated with pegloticase therapy, as well as the effect on tolerability. In part to expand clinical experience with pegloticase, we included among the 30 trial participants seven organ transplant recipients, a patient category at high risk for severe gout that was excluded from previous clinical trials [12]. As these patients require chronic immunosuppression to prevent organ graft rejection, we were interested in evaluating their susceptibility to developing Ab to pegloticase.

## Methods

### Materials

Pegloticase and 10 kDa mPEG (10 kDa *p*-nitrophenylcarbonate-activated monomethoxyPEG) were provided by Savient Pharmaceuticals (Bridgewater, NJ, USA). PEG-diol was obtained from Sigma-Aldrich (St. Louis, MO, USA), and PEGylated bovine adenosine deaminase (PEG-ADA) from Enzon Pharmaceuticals, Inc. (Piscataway, NJ, USA). Pegloticase was used under Investigator IND no. 11274 held by JSS.

### Study design

An open label trial was conducted at Duke University Medical Center to evaluate the ability of pegloticase infused every three weeks to maintain pUA below 6 mg/dL in 30 patients with refractory, symptomatic gout who were ≥18 years old and had serum uric acid concentration >7 mg/dL. Inclusion and exclusion criteria were similar to those in other trials of pegloticase [4,6,8,13], except for the participation of seven organ transplant recipients who were receiving immunosuppressive therapy to prevent graft rejection, and three patients who had received pegloticase in previous phase 1 or phase 2 trials >1 year prior to the present study. The trial protocol was approved by the Duke University Institutional Review Board, and all patients gave written informed consent.

The protocol called for five infusions of pegloticase (8 mg) at three-week intervals. Other urate-lowering therapy was discontinued 14 days prior to the trial. Unless contraindicated, colchicine or a non-steroidal anti-inflammatory drug was given as prophylaxis against gout flares. To minimize infusion reactions, patients received prednisone (20 mg) and fexofenadine (60 mg) orally the evening before each pegloticase infusion. Fexofenadine was repeated on the morning of the infusion, along with 200 mg of intravenous (i.v.) hydrocortisone.

### Pharmacokinetic and pharmacodynamic measurements

Pegloticase concentration in plasma (pUox) was monitored as uricase activity with a radiochemical high pressure liquid chromatography (HPLC) method [6,8], and is expressed as milliunits/mL plasma (1 Unit = 1 μmol of urate oxidized per minute at 37°C). Using this assay, each 8 mg dose of pegloticase contained approximately 145 U of uricase activity. Plasma urate concentration (pUA) in acidified plasma was measured by HPLC [6,8]. The precision for each assay was <5% coefficient of variation (CV) within the validated ranges, and the lower limits of quantification (LLOQ) for pUox and pUA were, respectively, 0.2 mU/mL and 0.5 mg/dL.

pUA and pUox were measured pre-treatment (Day 0) and at 23 points during treatment (at two hours and on days 7, 14 and 21 after each infusion; at 48 hours after infusions 1 and 5; and at a follow-up visit seven weeks after infusion 5. The plasma half-life (T_**½**_) for pegloticase was determined using all quantifiable pUox values, and at least three contiguous values were required. For determining the area under the concentration curve (AUC) for pUox and pUA, the LLOQ was used for values that fell below LLOQ.

The “average pUA during treatment” for each patient was the mean of all scheduled pUA measurements between Day 2 after infusion 1 and Day 21 after infusion 5 (nominally 21 measurements over 103 days). For patients who did not complete the study, the interval used was between Day 2 after infusion 1 and the last pUA measured within three weeks of the final infusion of pegloticase. The primary goal of treatment was an “average pUA during treatment”, as defined, of <6 mg/dL. Patients who had no more than one pUA >6 mg/dL during the observation interval were termed “persistent responders (PR)”; those who had two or more pUA values >6 mg/dL were termed “transient responders (TR)”. Non-response (NR) was defined as failure to achieve a pUA <6 mg/dL for any measurement.

### Immune response to pegloticase

Screening ELISAs to detect IgG Ab to both pegloticase and 10 kDa mPEG were scheduled for seven time points: at baseline, 21 days after each dose of pegloticase, and at the follow-up visit (nominally, days 0, 21, 42, 63, 84, 105 and 133). For patients who did not complete the trial, the final sample was obtained at their last visit. Following completion of the trial, some additional studies were performed to confirm epitope specificity and to better assess pre-existing Ab to PEG (described in Results). Aliquots of plasma that had been stored at -80°C were used for these studies.

#### Screening ELISAs (manual procedure)

Plasma samples diluted 1:21 in 1% BSA in PBS were tested by ELISA for IgG Ab to pegloticase and to 10 kDa mPEG essentially as described [6,8]. Cutoff values were the mean + 3 sd A405 obtained for a panel of 99 samples from normal donors: ≥0.178 with pegloticase, and ≥0.197 with 10 kDa mPEG. The positive QC and reference standard were from previously studied patients in whom pegloticase had induced high titer IgG Ab reactive with both pegloticase and 10 kDa mPEG, but not with unmodified uricase [6,8]. Prior to use, positive QC was run a minimum of 20 times and ranges were calculated; this QC was then used to assess the quality of subsequent assays. The screening ELISAs were validated according to published guidelines for precision, specificity, recovery, interference, linearity and range, stability and ruggedness. Intra-assay precision was ≤7.1% CV with pegloticase as the plate coat, and ≤11.4% CV with 10 kDa mPEG; inter-assay precision was <9% CV and <12.1% CV, respectively.

#### Automated ELISA

The manual screening procedure was modified to use a Tecan Evo 75 liquid handler and an automated plate washer (Tecan Mannedorf, Switzerland). As in the screening ELISA, bound human IgG was detected with goat anti-human IgG conjugated to alkaline phosphatase, using p-nitrophenyl phosphate as the substrate. However, the reaction was stopped at 20 minutes with 10% NaOH, rather than when the positive standard reached an A405 of about 1. The automated procedure had a lower background and was somewhat more sensitive than the manual screening ELISA. During validation, the cutoff for the automated ELISA was determined to be A405 ≥ 0.15.

#### Competition ELISA (automated procedure)

Plasma samples stored at -80°C were thawed and diluted 1:21 into 1% BSA in PBS, alone or containing 200 μg/mL of either 10 kDa PEG-diol (that is, PEG terminated with neither a methoxy group nor an activated linker) or un-modified recombinant porcine uricase. After incubating at 4°C overnight, 0.1 mL aliquots (containing no competitor or 20 μg of either PEG-diol or uricase) were tested in duplicate for IgG Ab to pegloticase, using the automated ELISA procedure. A decrease of >32% in A405 (compared to no competitor) indicated specificity for the competing antigen.

### Evaluation of clinical response and safety

At clinic visits prior to each infusion and at follow-up, the following were performed: a count of tenderness and swelling of 68 joints; the number of flares experienced by patients or assessed by study personnel were recorded; patient and physician global assessment were assessed on a visual activity scale; and functional status was assessed by Health Assessment Questionnaire (HAQ). Photographs of hands were obtained at baseline and at the final follow-up clinic visit.

### Statistics

Except as indicated, data are expressed as mean ± SD. Statistical analyses were performed using JMP software, version 9.0 (SAS Institute, Cary, NC, USA). Fisher’s exact test was used to assess the association between two kinds of categorical data. The correlation between responses in ELISAs using different immobilized antigens was assessed by ANOVA and linear regression. Means for patient subgroups were compared by ANOVA. *P*-values of <0.05 were considered to be significant.

## Results

Of 42 patients screened, 12 withdrew prior to dosing. The 30 who entered the trial had severe gout and commonly associated co-morbidities (Table 1). Among these patients, 27 were naïve to pegloticase and 3 had received pegloticase in previous clinical trials. Two of the latter patients had received single doses of 1 mg and 12 mg in phase 1 trials [6,8], and the third had received multiple infusions (information about dose and number of infusions unavailable) in a phase 2 trial [13]. All three of these patients had developed antibodies to pegloticase following their initial exposure, which had occurred one to three years before re-exposure in the present trial. Among the 27 pegloticase-naïve patients, 7 were organ transplant recipients, who were receiving various immunosuppressive regimens to prevent graft rejection (Table 2).

**Table 1:** Demographics

Gender	8 F, 22 M
Age, years	57 (25 to 93)
Baseline pUA, mg/dL	10.8 ± 1.2 (9.0 to 13.7)
Tophi, chronic gout arthritis	28 (93%)
Wt, kg	95.0 ± 31.2 (44 to 203)
BMI	31.5 ± 7.5 (19 to 56)
Serum creatinine, mg/dL	1.9 ± 0.7 (0.8 to 3.2)
**Most frequent comorbidities**	
Osteoarthritis	4 (13%)
Hypertension	27 (90%)
Diabetes	12 (40%)
Cardiovascular disease	14 (47%)

BMI, body mass index; pUA, plasma urate concentration.

**Table 2:** Organ transplant recipients: immunosuppressive therapy

**Patient ID (age)**	**Organs transplanted**	**Immunosuppressive regimen**	**Year initiated**
203 (65 y)	kidney	Mycophenolate mofetil 500 mg QD	1995
207 (45 y)	kidney	Cyclosporine 175 mg BID; Azathioprine 100 mg QD	1994
208 (32 y)	kidney + pancreas	Mycophenolate mofetil 500 mg BID; Tacrolimus 2 mg BID	2000
211 (47 y)	kidney + pancreas	Cyclosporine 150 mg BID; Azathioprine 50 mg QD	1991
214 (55 y)	kidney	Cyclosporine 125 mg TID	1991
233 (41 y)	kidney x 2	Cyclosporine 75 mg BID; Azathioprine 50 mg QD	1993*
238 (51 y)	kidney	Cyclosporine 50 mg BID; Mycophenolate mofetil 1000 mg BID	1999

*Year of second kidney transplant. The regimen taken between 1988 (year of first kidney transplant) and 1993 was not recorded.

One pegloticase-naïve patient was withdrawn from the study before completing the first infusion of pegloticase due to a syncopal reaction. He was classified as a non-responder (NR, see Methods), and was included in the analysis of safety, but not of pharmacokinetics (PK) and pharmacodynamics (PD).

### Pharmacokinetics and pharmacodynamics

**Figure 1:**
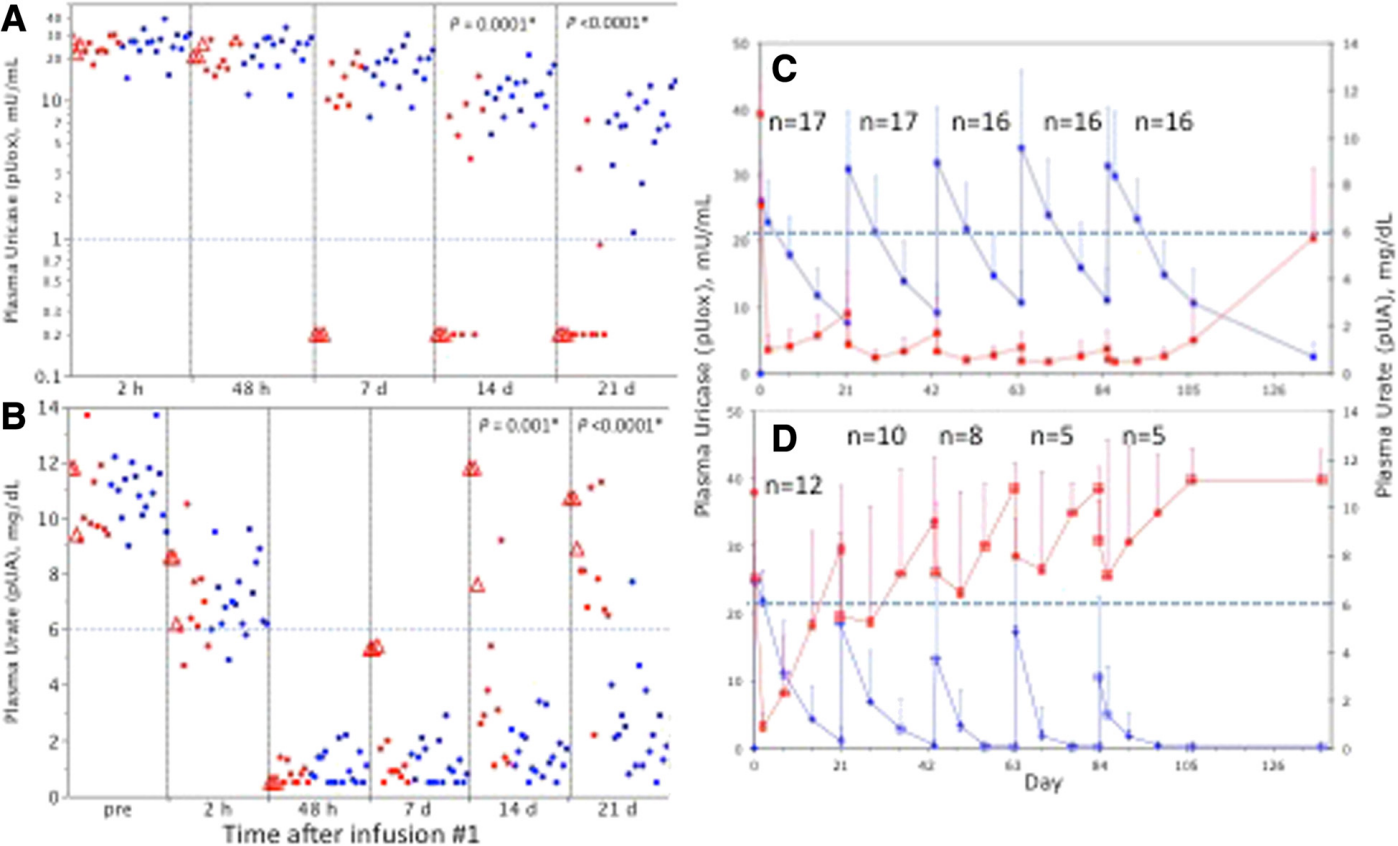
**Pharmacokinetics and pharmacodynamics of pegloticase infused at three-week intervals. ***Panels ****A ****and ****B***: Data for the first pegloticase dosing cycle on all 29 patients. **(A)** Plasma uricase activity (pUox), plotted on a logarithmic scale (y-axis). The dashed line at 1 mU/mL is shown as a reference. **(B)** Plasma uric acid concentration (pUA). The dashed line at 6 mg/dL is shown as a reference. Symbols: blue = patients with a persistent response to pegloticase (PR); red = patients with a transient response to pegloticase (TR); open red triangles = 3 non-naïve TR, who had received pegloticase >1 year prior to the present trial. *P-*values shown are for comparison of the means for PR and TR. *Panels ****C**** and ****D***: Mean pUox and pUA during all five dosing cycles for PR **(C)** and TR **(D)**. Symbols: blue circles = pUox; red squares = pUA; n = number of patients under treatment in each cycle. Error bars, standard deviation.

Data for pUox and pUA are plotted in Figure 1; PK and PD parameters derived from these data are listed in Table 3. In overview, pUA normalized rapidly in all 29 treated patients, and remained <6 mg/dL in 17 persistent responders (PR), of whom 16 (94%) completed the trial. Hyperuricemia recurred in 12 transient responders (TR), of whom 5 (42%) completed the trial. These response patterns became evident during the first dosing cycle (Figure 1A, B), but they were not predicted by differences in any baseline parameters (Table 1), including gender (*P* = 0.41), age (*P* = 0.67) or body weight (*P* = 0.74).

**Table 3:** Summary of pharmacokinetic parameters

***Patients***	**All**	**PR**	**TR**	***P*, PR vs. TR**
***Infusion #1***				
C_max_ (t = 2 h) mU/mL	25.6 ± 5.0	26.2 ± 5.9	24.7 ± 3.4	0.45 (ns)
C_48h_ (t = 48 h) mU/mL	22.4 ± 5.6	22.9 ± 6.3	21.7 ± 4.7	0.60 (ns)
C_min_ (t = 21 days) mU/mL	4.9 ± 4.6	7.7 ± 3.8	1.0 ± 2.2	<0.0001*
T_1/2_, days	11.4 ± 3.7^a^	12.3 ± 3.7	9.7 ± 3.3^a^	0.08 (ns)
AUC_2 h-21 days_, mU*mL^-1^*d	277 ± 112	328 ± 100	204 ± 85	0.0016*
***Infusion #5***				
C_max_ (t = 2 h) mU/mL		31.4 ± 9	10.5 ± 12	<0.0005*
C_48h_ (t = 48 h) mU/mL		29.9 ± 9.7	4.9 ± 7.3	<0.0001*
C_min_ (t = 21 days) mU/mL		10.6 ± 5.1	0.2	<0.0003*
T_1/2_, days		13.8 ± 3.0	-^b^	
AUC_2 h-21 days_, mU*mL^-1^*d		422	44	
AUC, inf #5/inf #1		1.3	0.2	
***Average for all infusions***				
C_max_ (t = 2 h) mU/mL		30.9 ± 2.9	16.9 ± 5.4	0.0009*
C_min_ (t = 21 days) mU/mL		9.9 ± 1.4	0.4 ± 0.4	<0.0001*
T_1/2_, days		13.0 ± 3.4	-^b^	
AUC_2 h-21 days_, mU*mL^-1^*d		397 ± 38	105 ± 65	<0.0001*

^a^T_1/2_ could not be calculated for three non-naive patients; therefore N = 26 (all), 17 (PR), 9 (PR); ^b^T_1/2_ could not be calculated. AUC, area under the concentration curve.

By 48 hours after infusion 1, mean pUox for all 29 patients had declined by 12.5% from the level at two hours, and mean pUA had fallen to 1.0 ± 0.6 mg/dL, a 91% decrease from baseline (Figure 1A, B). By Day 7, pUox had become undetectable and pUA had risen to almost 6 mg/dL in the three TR with prior exposure to pegloticase. Over the next two weeks, eight of the nine pegloticase-naïve TR also lost response. On Day 21, pUox was measureable in all 17 PR, but in only 3 of 12 TR; mean pUox for the PR and TR was 7.7 ± 3.8 and 1.1 ± 2.1 mU/mL, respectively (*P* <0.0001). Conversely, pUA exceeded 6 mg/dL in 11/12 TR vs. 1/17 PR; mean pUA was 8.3 ± 2.6 for TR and 2.6 ± 1.8 mg/dL for PR (*P* <0.0001).

Mean PK and PD data for all five dosing cycles are plotted in Figure 1C, D. For PR (Figure 1C), the PK pattern in each cycle was consistent, with C_max_, C_48h_, C_min_ and AUC for pUox increasing in successive cycles to approach steady state by the fifth cycle. For each parameter, the ratio of the value in cycle 5 to that in cycle 1 was 1.2 to 1.4 (Table 3). This accumulation is consistent with a T_1/2_ of 13.0 days (mean for all cycles for the 16 PR who received all five doses; T_1/2_ in cycle 5 was 13.8 days). The AUC for pUA showed a reciprocal decline in successive cycles, yielding a ratio of cycle 5/cycle 1 of 0.4. The average pUA during treatment (defined in Methods) for all PR was 1.0 ± 0.4 mg/dL. At the follow-up visit seven weeks after the last dose, mean pUA for the 16 PR was 5.7 ± 3.0 mg/dL.

TR continued to lose response to pegloticase beyond cycle 1, and only 5 of 12 received the last two infusions (Figure 1, panel D). The average pUA during treatment for all TR was 7.5 ± 2.6 mg/dL. For the five who completed the trial, the ratio of AUC in cycle 5 to cycle 1 was 0.2 for pUox, and 2.4 for pUA; at follow-up, mean pUA was 11.1 ± 1.4 mg/dL.

### Immune response to pegloticase

**Figure 2:**
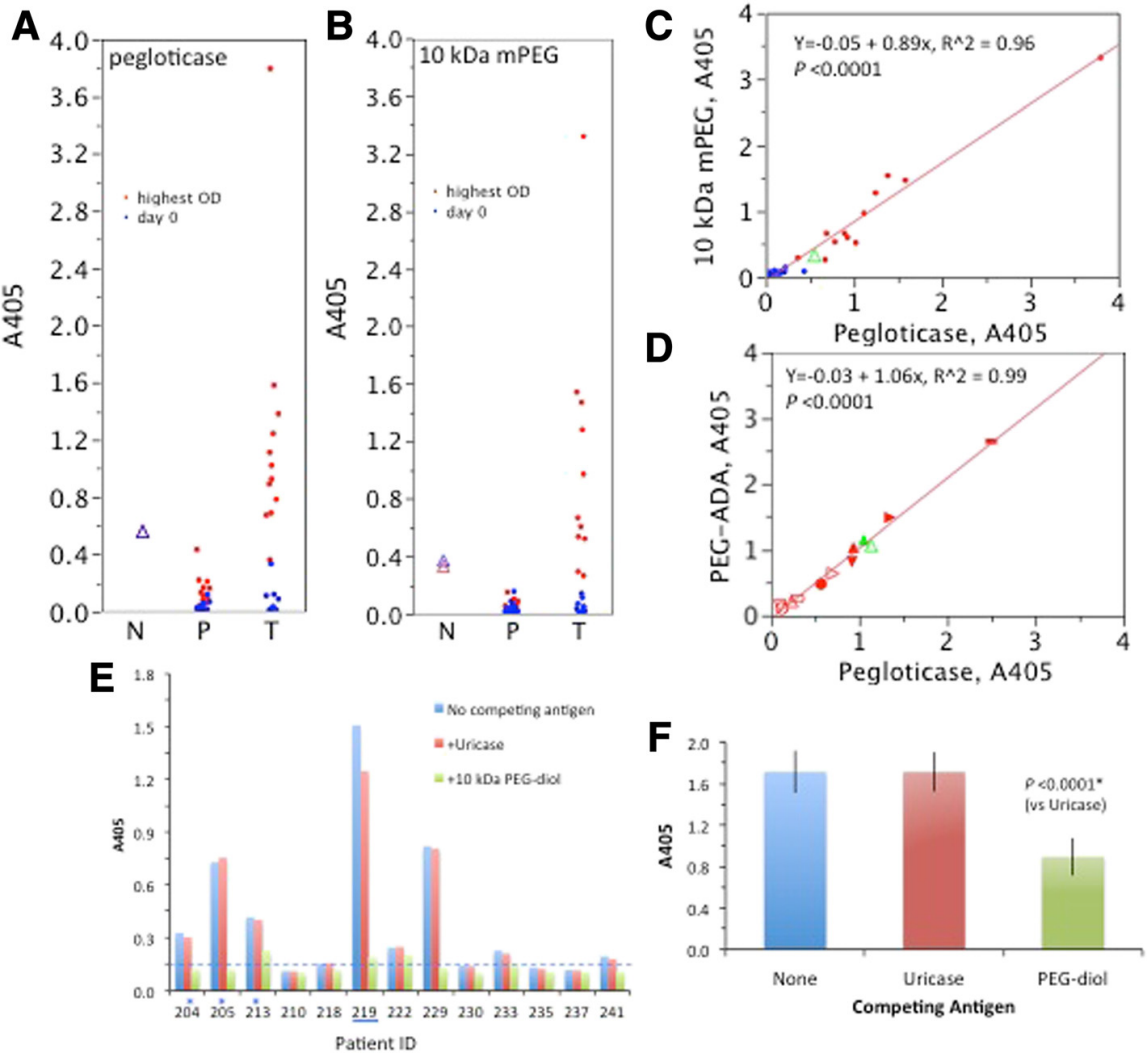
**Antibody response to pegloticase.** Screening ELISAs for Ab to pegloticase **(A)** and10 kDa mPEG **(B)**. Horizontal axis: N = non, P = persistent, T = transient responder. Symbols: blue = pre-treatment; red = highest A405 during the trial; triangles = the non-responder. **(C)** Correlation between the anti-pegloticase and anti-10 kDa mPEG ELISAs. The highest A405 values during treatment from 2A and B are re-plotted. Symbols: blue = persistent (P), red = transient (T), green = non-responder (N). **(D)** Correlation between ELISAs for Ab to pegloticase and PEGylated adenosine deaminase (PEG-ADA). Conditions for the ELISAs were the same except for the plate coating. Two samples, obtained on Day 0 (open symbols) and at the final visit (filled symbols) from six patients (distinguished by symbol shape) were studied. Competition ELISA (see Methods). **(E)**. Pre-treatment. The non-responder (blue underline) and three previously treated patients (blue asterisks) are indicated on the horizontal axis. **(F)**. Post-treatment. The last sample obtained from each Ab-positive patient was tested to minimize competition from circulating pegloticase (pUox was undetectable or <0.3 mU/mL). Data plotted are the mean and SEM for the 13 samples tested.

Figure 2A, B plots the baseline and the highest A405 values attained during treatment in screening ELISAs for Ab to pegloticase and 10 kDa mPEG. Prior to treatment (blue symbols), most patients were negative or borderline positive in both screens. Of note, the single NR had the highest pre-treatment response to both antigens. During treatment (red symbols), 3 of 17 PR developed positive screens on six occasions (5.2% of 115 samples tested). A405 in the anti-pegloticase screen exceeded 0.4 on only one occasion in one patient. By contrast, all 12 TR developed Ab to both antigens; 47 of 62 (75.8%) samples tested were positive, and the mean of the highest A405 values in both ELISAs was 20-fold higher than the mean for Day 0 (*P* <0.0001). In other studies, IgG anti-pegloticase Ab titers in TR ranged from approximately 1:100 to >1:12,000, and, as observed during phase I testing, the predominant IgG subclasses were IgG2 and IgG3 [6,8]. In mixing experiments, Ab-positive plasma samples did not appreciably inhibit pegloticase enzymatic activity (that is, Ab were not neutralizing).

As noted, all TR developed Ab to both pegloticase and 10 kDa mPEG. That the same Ab recognizes both antigens is suggested by the strong correlation between A405 values in the two ELISAs (Figure 2C), and between A405 values obtained in ELISAs using pegloticase and PEG-ADA as plate coatings (Figure 2D). PEG is the only common feature of these two entities: in PEG-ADA, 5 kDa mPEG is linked to bovine ADA via a succinyl ester, whereas in pegloticase, 10 kDa mPEG is attached to porcine uricase by a one carbon urethane bond.

In addition to testing recognition of immobilized antigens, we performed a competition ELISA to interrogate Ab binding to antigens in solution (Figure 2E, F). Spiking Ab-positive plasma samples with 10 kDa PEG-diol (which lacks a methoxy group), but not with un-PEGylated uricase, caused significant inhibition of the anti-pegloticase ELISA. Inhibition by PEG, but not uricase, was observed with pre-treatment samples from 8 patients (Figure 2E), and with post-treatment samples from all 13 Ab-positive patients (Figure 2F). Considering all results in Figure 2, we conclude that Ab to pegloticase are directed at internal ethylene oxide units of PEG, rather than to the uricase protein, the methoxy terminus of mPEG, or the protein-polymer junction.

**Figure 3:**
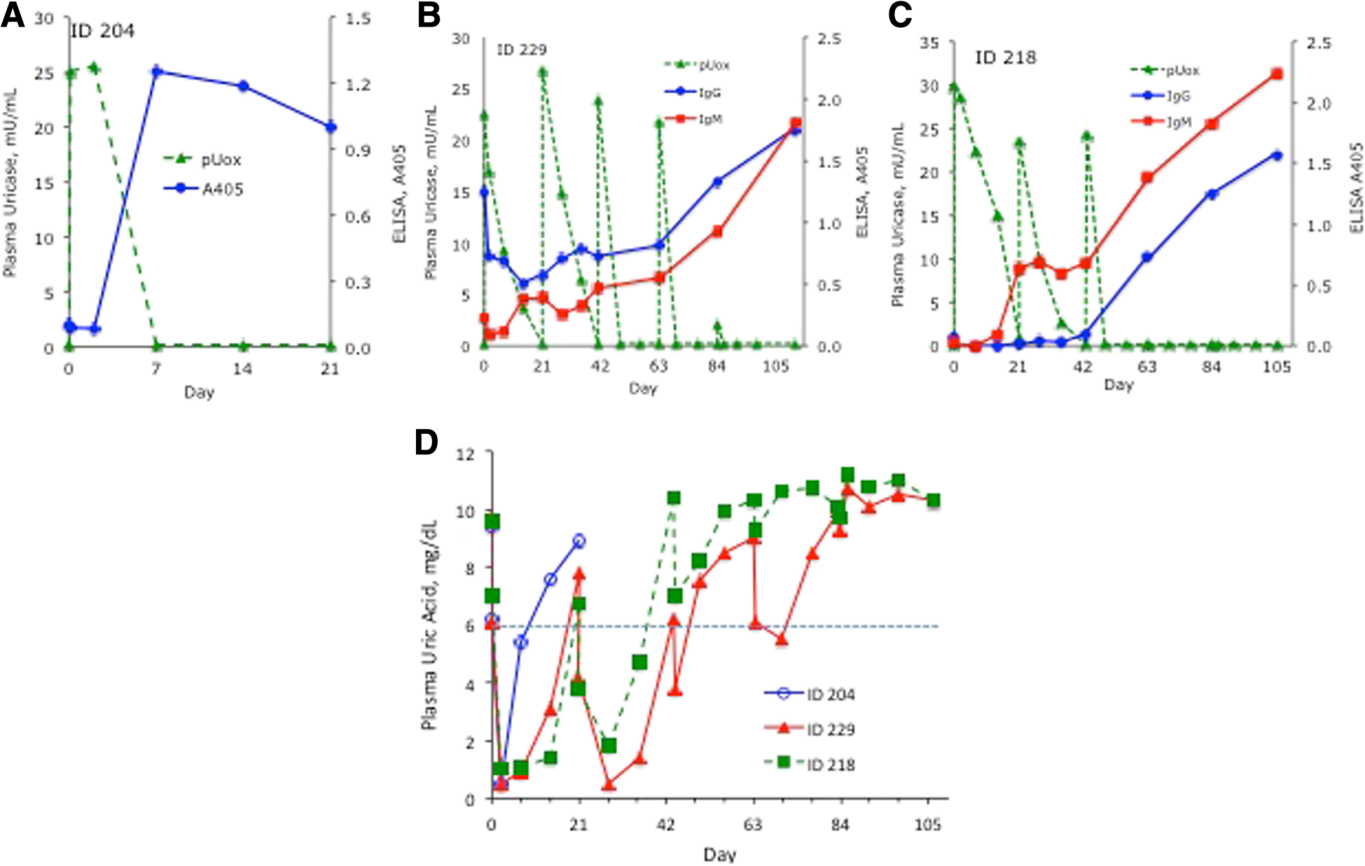
**Evolution of Ab to pegloticase and relation to pUox and pUA in three patients with a transient response to treatment with pegloticase. (A-C)** Change during treatment in pUox (left vertical axis) and in the ELISAs for Ab to pegloticase (right vertical axis). **(D)** Change during treatment in pUA for the same three patients.

The evolution of Ab to pegloticase varied among patients, presumably due to factors that can influence the timing of induction of immunity (for example, prior exposure to antigen), and the sensitivity of Ab detection (for example, the avidity of Ab for circulating antigen). Figure 3 illustrates patterns for three patients. In patient 204 (Figure 3A), who had received pegloticase two years earlier, IgG Ab increased sharply between days 2 and 7 after re-exposure, coincident with elimination of pUox (he received no further treatment). The other two patients were naïve to pegloticase and received all five infusions. In patient 229 (Figure 3B), IgG and IgM Ab to pegloticase were present in pre-treatment plasma. Both isotypes decreased after the first dose of pegloticase, then recovered during dosing cycles 2 and 3, and rose further as pUox declined in cycles 4 and 5. In patient 218 (panel 3C), IgM Ab to pegloticase appeared two weeks after infusion 1, preceding IgG Ab by four weeks. Both isotypes rose more rapidly after pUox became un-measureable in cycle 3. pUA levels in each of these three patients mirrored the fluctuation in circulating pUox (Figure 3D).

Of interest, among the 27 pegloticase-naïve patients, only 1 of 7 (14%) organ transplant recipients developed Ab to pegloticase or 10 kDa mPEG, vs. 9 of 20 (45%) non-recipients. One of the six Ab-negative transplant recipients received only two doses of pegloticase, but at the point she was last tested for Ab (Day 42), all nine non-recipients had already developed Ab.

### Safety and tolerability

As in previous trials of pegloticase, the most common adverse events were gout attacks, experienced by 27/30 patients (90%), and infusion reactions, which occurred in 13/30 patients (43%). Among Ab-positive patients, 8 of 13 (62%) experienced reactions during 10 of 41 (24%) infusions; 5 withdrew from the trial because of these reactions, which are summarized in Table 4. Among Ab-negative patients, only 5 of 17 (29%) had reactions associated with 11 of 82 (13%) infusions. These reactions were treated by slowing or stopping the infusion of pegloticase and administering diphenhydramine; when the reaction resolved, infusion of pegloticase was resumed at a slower rate. None of these reactions led to the discontinuation of treatment.

**Table 4:** Infusion reactions leading to withdrawal from study

**Patient ID (age)**	**Number infusions**	**Reactions**	**Treatment/outcome**
210 (50 y)	2	Nausea, dyspnea, hypoxemia, tachycardia, diaphoresis, hypotension, systemic rash	IV fluids, O_2_, epinephrine, diphenhydramine; hospitalized overnight. All symptoms resolved.
213 (43 y)	3	Chills, nausea, chest tightness, systemic urticaria	Infusion slowed, acetaminophen, diphenhydramine. All symptoms resolved.
219 (67 y)	<1	Syncope	Infusion stopped. All symptoms resolved.
237 (25 y)	3	Chest tightness, dyspnea, flushing, tachycardia	Infusion stopped; diphenhydramine. All symptoms resolved.
241 (70 y)	2	Pruritus, chest tightness, hypotension, hypoxemia	Infusion stopped; IV fluids, O_2_, diphenhydramine. All symptoms resolved.

**Figure 4:**
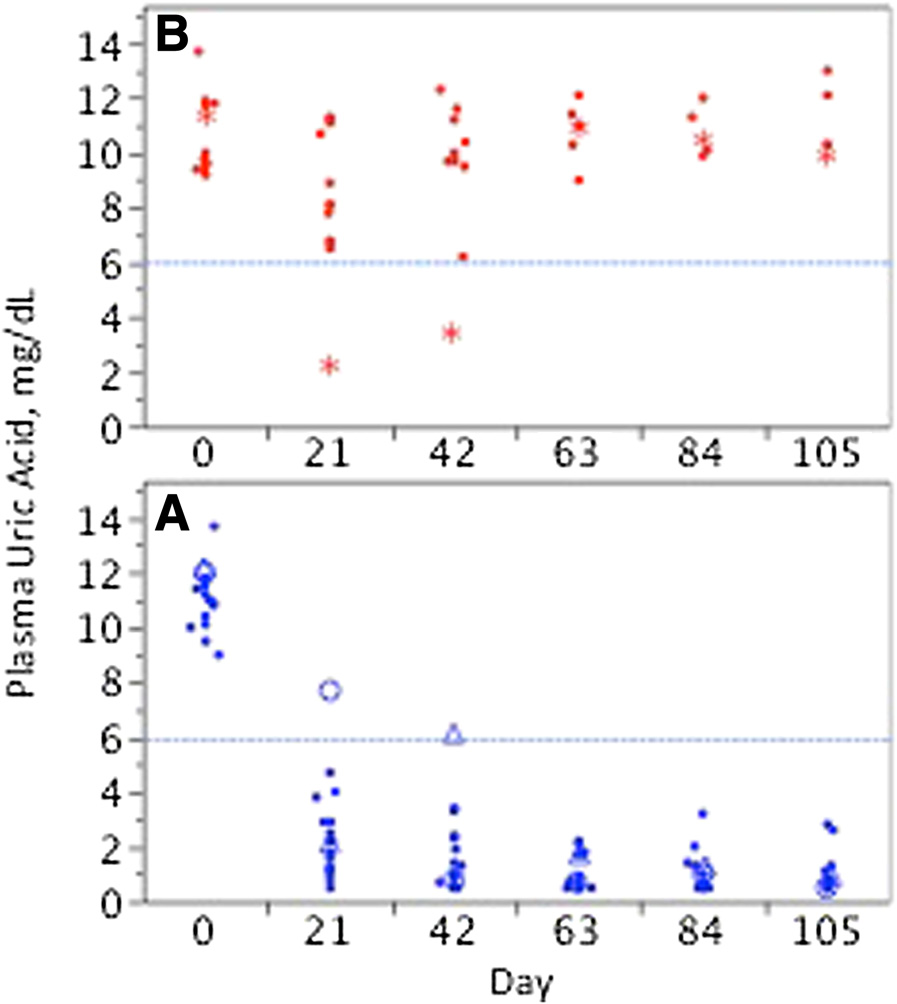
**Pre-infusion (trough) pUA as a marker for a significant antibody response to pegloticase.** All pUA determinations obtained immediately prior to each infusion of pegloticase (nominally 21 days after the preceding infusion) are plotted. The dashed line at 6 mg/dL is shown as a reference. **(A)** Persistent responders (blue symbols). The open circles and open triangles represent data for the two PR who had single pre-infusion (trough) pUA values >6.0 mg/dL. **(B)** Transient responders (red symbols). The asterisks represent data for the single TR in whom any pre-infusion (trough) pUA <6 mg/dL was observed.

As a strategy for minimizing infusion reactions in patients receiving 8 mg of pegloticase every two weeks, it has been recommended that if pUA measured prior to infusions exceeds 6 mg/dL on two consecutive occasions, discontinuation of treatment should be considered [4,5]. This strategy is also reliable in marking the development of anti-PEG Ab and loss of efficacy in patients receiving the same dose every three weeks. Thus, as shown in Figure 4A, among all PR, trough (pre-infusion) pUA was >6 mg/dL on only single occasions in 2 of 17 patients (7.7 mg/dL after infusion 1 in one case, and 6.1 mg/dL after infusion 2 in the other). By contrast all trough/pre-infusion pUA values were >6 mg/dL in 11 of 12 TR, and after infusions 3 to 5 in the 12^th^ patient (Figure 4B).

Four patients experienced serious adverse effects (SAEs). There was no discernible common mechanism, and no relationship to infusions of pegloticase. A patient with diabetes mellitus type 2 required hospitalization for hyperglycemia, and a patient receiving warfarin was hospitalized for a gastrointestinal bleed; both went on to complete treatment and were PR. The other two SAEs occurred in organ transplant recipients. A 32-year-old female with type 1 diabetes mellitus, who was one of two kidney/pancreas recipients, had a duodenal perforation during her second dosing cycle, which led to loss of her pancreas graft and withdrawal from the trial. She was negative for anti-PEG Ab and had a pUA of 0.9 mg/dL at three weeks after her last dose of pegloticase. A 41-year-old man who had twice received renal transplants for Alport’s hereditary nephritis, had a fatal myocardial infarction nine days after his third dose of pegloticase. He had known coronary artery disease and other cardiac risk factors (hypertension, renal insufficiency, obesity, obstructive sleep apnea). Anti-PEG Ab developed after one dose of pegloticase, and his average pUA during treatment was 8.3 mg/dL; his pUA at a scheduled clinic visit two days before the fatal event was 11.6 mg/dL.

**Figure 5:**
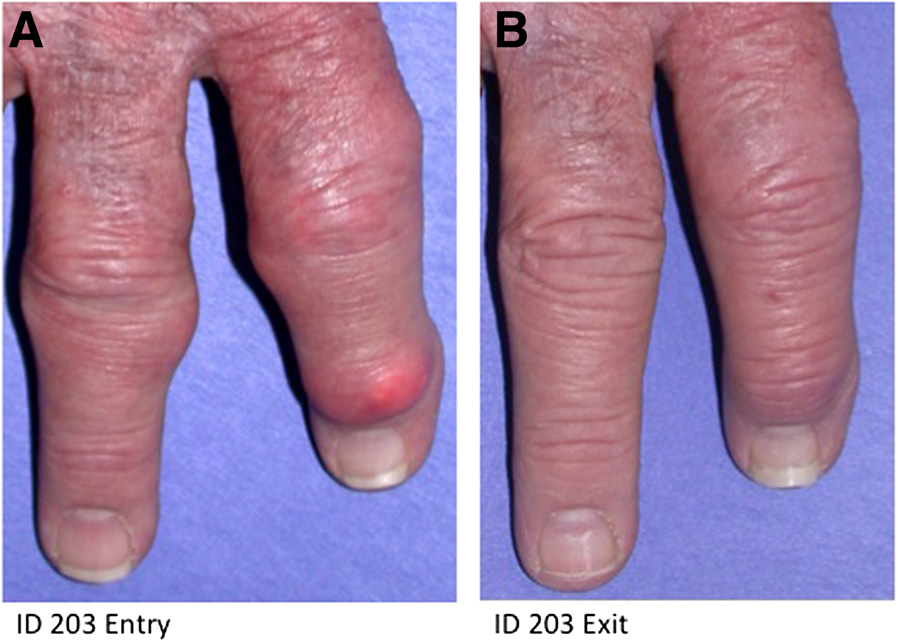
**Resolution of digital tophi in a persistent responder. (A).** Prior to treatment with five doses of pegloticase, 8 mg, infused at three-week intervals. **(B).** At follow-up visit.

Because the current trial was short-term and open label, the ability to assess benefit was limited. However, for the 21 patients who completed the trial, five measures of clinical response showed improvement when observations at week 19 (seven weeks after the final infusion) were compared with baseline (Table 5). For four of the five parameters, the median percentage improvement in PR exceeded that for TR by 1.6- to 7.2-fold (tender joint count, patient pain, and both physician and patient global assessment). The sustained reduction in pUA to <2 mg/dL in PR was in some cases accompanied by reduction in size or complete resolution of digital tophi, as illustrated in Figure 5. Resolution of tophi in another PR patient was reported elsewhere, in an analysis of oxidative stress status in patients in this clinical trial [7].

**Table 5:** Clinical response measures

	**Median day 0 (n = 30)**	**Median day 134 (n = 21)**	**Median improvement persistent responders (n = 16)**	**Median improvement transient/non-responders (n = 5)**
Tender joint count	13	2	70%	44%
Swollen joint count	9	6	49%	40%
Patient global assessment	44.0	9.0	67%	25%
Patient pain	52.0	23.0	65%	9%
Physician global assessment	45.5	24.5	61%	20%

## Discussion

### Efficacy of every three-week dosing

Infusing 8 mg of pegloticase every three weeks controlled hyperuricemia in 17 of 30 patients. In the 16 PR who received five doses, pUA averaged approximately 1 mg/dL, a decrease of >90% from baseline, during the 15-week treatment period. This is comparable to pUA achieved over the same period in patients classified as “responders” in phase 3 testing, who received 8 mg of pegloticase every two weeks [4].

Two points regarding PK and PD data in persistent responders are noteworthy. Control of pUA in PR was unrelated to body weight (44 to 203 kg), and Cmax, Cmin and AUC for pUox were approximately 30% higher during the fifth dosing cycle than during the first. This “positive accumulation” reflects the 13-day half-life of pegloticase, and is consistent with PK modeling [14]. These PK results, combined with the very low pUA maintained, indicate that plasma uricase activity provided by 8 mg of pegloticase must approach or exceed uric acid production over a three-week dosing interval (for further discussion, see [7]). A very low pUA mobilizes urate from tophi, and maintaining uricase in plasma throughout the dosing cycle ensures its degradation. This is important in refractory gout patients, in whom urate excretion is often impaired.

Given the short trial period and patient population, it was gratifying to observe meaningful improvement in pain, tender joint count and global assessment of disease activity, as well as resolution or reduction in tophus size in some patients. Almost all patients experienced gout flares, but this was expected given the indication for treatment, and because the frequency of flares tends to increase with initiation of all urate-reducing therapies. Maintaining a very low pUA with continued treatment would be expected to progressively reduce both gout flares and tophus size [15].

### Anti-PEG Ab response

None of the 17 PR mounted a significant or sustained Ab response to pegloticase. In the other 13 of 30 patients (43%), Ab to pegloticase was associated with a rapid decline in pUox and increase in pUA. These events occurred between days 2 and 7 after the first infusion in the three patients with a prior exposure to pegloticase, and over the next two weeks in eight of nine pegloticase-naïve TR. Ab to pegloticase doubled the risk of infusion reactions; 5 of 13 Ab-positive patients withdrew from the trial due to these reactions, vs. 0 of 17 Ab-negative patients.

Similar to the present results, during phase 1 testing, single doses of pegloticase (0.5 to 12 mg) induced IgG and IgM anti-pegloticase Ab within three weeks in 38% of patients [6,8]. In previous phase 2 and 3 trials lasting up to six months, Ab to pegloticase was detected (using different methods) at some point in >80% of patients; the highest titers were preferentially associated with loss of efficacy and infusion reactions [4,13].

Epitope specificity was not addressed in reports of previous phase 2 and 3 trials. Our present findings add to evidence obtained during phase 1 testing by demonstrating that Ab to pegloticase recognize the ethylene oxide backbone of PEG, rather than the uricase protein, the linker joining PEG to uricase, or the methoxy terminus of mPEG. Our findings do not support speculation that Ab with high affinity for methoxy groups contribute to the loss of efficacy of pegloticase [16]. With longer treatment, some patients might develop Ab to the uricase protein. However, the initial Ab formed, which are responsible for rapid clearance of pegloticase and loss of efficacy, are directed at PEG.

It is noteworthy that anti-PEG Ab were detected in pre-treatment plasma from eight patients. Three of these patients had previously received pegloticase, but five had not; they represented 19% of the 27 pegloticase-naïve patients, and half of the 10 non- or transient responders. Pre-existing anti-PEG Ab may have resulted from exposure to “free” (that is, unconjugated to protein) PEG-diols, or various ether and fatty acid derivatives of PEG, which have been used for decades as solubilizers, stabilizers or excipients in cosmetics, laxatives and medicinal preparations. The general prevalence of anti-PEG Ab, and factors that may be responsible for their development, is a subject we are pursuing.

A recent “critical review” citing flawed or un-validated methods has questioned the induction of anti-PEG Ab by PEGylated therapeutic agents in humans, and has argued that biological effects attributed to anti-PEG Ab “lack the characteristics of a bona fide Ab reaction” [11]. Our present findings, obtained with validated methods, unequivocally demonstrate that anti-PEG Ab are relevant to pegloticase therapy. The temporal relationship between exposure to the PEGylated antigen and anti-PEG Ab induction, including an accelerated response upon re-exposure to pegloticase, are entirely consistent with a “bona fide Ab reaction”. Much remains to be learned about how the immune system recognizes and responds to PEG, which has physical and chemical properties very different from protein and other polymeric antigens.

### Treatment of organ transplant recipients with pegloticase

Managing gout in organ transplant recipients is complicated by serious underlying diseases, and by drug regimens needed to treat these conditions and to prevent graft rejection [12]. We felt that these patients could benefit from the ability of pegloticase to rapidly deplete urate deposits, even when urate excretion is impaired, while their chronic immunosuppression might dampen the immune response to pegloticase. Five of 7 transplant recipients did respond well to pegloticase, and only 1 of 7 developed Ab to pegloticase, vs. 9 of 20 pegloticase-naïve non-transplant recipients. Although the number of patients is small, this difference supports the investigation of immunosuppressive strategies as an adjunct to pegloticase therapy.

The burden of chronic disease carried by organ transplant recipients is underscored by SAEs in two patients, especially a fatal myocardial infarction in a 41-year-old renal transplant recipient with hereditary nephritis. Anti-PEG Ab had developed rapidly in this patient, and he was markedly hyperuricemic during the month prior to his myocardial infarction. In addition to his multiple other cardiac risk factors, gout and hyperuricemia independently predict mortality in patients with cardiovascular disease [17-21]. The risk may be greatest in patients with chronic tophaceous gout and a high level of hyperuricemia [22].

## Conclusions

Infusing 8 mg of pegloticase every three weeks is consistent with PK and effectively controlled hyperuricemia in 17 of 30 study patients, and five such infusions led to the amelioration of symptoms due to gout. Whether or not every three-week dosing is used routinely, physicians and patients may be reassured by our findings that missing an occasional every two-week dose of pegloticase will not result in loss of control. In patients who have had a good response to an every two-week schedule, switching to every three-week dosing may be an option, particularly for patients who are frail or must travel a distance to an infusion facility. In 13 of 30 patients, Ab to pegloticase that recognize PEG rather than the uricase protein caused the rapid elimination of pegloticase from plasma, associated with loss of efficacy and a doubled risk of infusion reactions. Anti-PEG Ab usually appeared after the first dose of pegloticase, and were pre-exisiting in 5 of 10 non- or transient responders who were naïve to pegloticase. Two consecutive trough (pre-infusion) pUA values >6 mg/dL were reliable markers for anti-PEG Ab and loss of efficacy. Pegloticase can be effective, and may be less immunogenic in organ transplant recipients with refractory gout; the risks and benefits in these complex patients should be carefully considered. Our findings suggest that an immunosuppressive strategy should be investigated as a means of reducing the antibody response to pegloticase.

## Abbreviations

Ab: Antibody/antibodies; ADA: Adenosine deaminase; AUC: Area under the concentration curve; BSA: Bovine serum albumin; ELISA: Enzyme linked immunosorbent assay; HPLC: High pressure liquid chromatography; LLOQ: Lower limit of quantification; mPEG: Monomethoxy-polyethylene glycol; NR: Non-responder; PBS: Phosphate-buffered saline; PD: Pharmacodynamics; PEG: Polyethylene glycol; PEG-ADA: PEGylated bovine adenosine deaminase; PK: Pharmacokinetics; PR: Persistent responder; pUA: Plasma uric acid concentration; pUox: Plasma uricase activity; SAEs: Serious adverse effects; TR: Transient responder.

## Competing interests

MSH and JSS have on occasion been paid as consultants to Savient Pharmaceuticals. Duke University, MSH and SJK, and Mountain View Pharmaceuticals, Menlo Park, CA, hold patent rights in pegloticase and its use, which have been licensed to Savient Pharmaceuticals. Duke University, MSH and SJK receive royalties from the sales of pegloticase.

## Authors’ contributions

MSH and JSS contributed equally to the design of the clinical trial protocol. MSH was Principle Investigator of the FDA Office of Orphan Product Development Grant that provided major support for the clinical trial. JSS was Principle Investigator of the clinical trial, and ES and DJ were clinical coordinators. JSS, ES and DJ were responsible for screening and obtaining informed consent from patients, and for acquiring and evaluating data related to clinical response and adverse events. NJG and SJK performed, and with MSH analyzed the biochemical and immunological studies reported. MSH drafted the manuscript, which was then reviewed and revised critically for intellectual content by NJG, SJK, ES, DJ and JSS. All authors gave final approval for submitting the manuscript for review.
